# The Neutral Treatment in Fevers

**Published:** 1884-08-20

**Authors:** L. L. Holcombe

**Affiliations:** Terrebonne Station, La.


### THE NEUTRAL TREATMENT IN FEVERS.

BY DR. L. L. HOLCOMBE, TERREBONNE STATION, LA.

After a long experience and observation in the treatment of fevers of all grades and characters, I have found, as an almost invariable thing, a strictly neutral system to be the best. This idea was first suggested to me years since, when I was a student of medicine. My reading taught me that there are few medicines of the materia medica which have maintained a lasting reputation for the cure of fevers that are not alkalies or alkaloids; *e. g.*, the cinchona alkaloids, gelsemium, arsenic, the neutral salts, etc., etc. These are powerful antipyretics, as everybody knows. Now, why so. They act by neutralizing the alimentary canal. They destroy zymotic fermentation by their neutralizing powers simply. In fact, most medicines that are supposed to possess antipyretic powers are peculiarly destructive to fermentation.

In short, our theory of fever is, in reality, based upon the now generally accepted belief that organic germs, which originate in fermentative processes, propagated by natural cultivations in the *primæ viæ* and in the circulating juices of the system, are the prime causes of fevers of every description and class. The great genius of Broussais, Brown and Rasori lit the first sparks that have thrown a refulgent light upon the causes of fever. Upon their theories and notions (be it admitted or not) many of our views of fever and its causes are now based. But as we are not presuming to write a commentary, we will strictly confine ourselves to the subject heading of this article.

Acids of every class and description are prejudicial in fever. Though antipyretic in a sense, on account of their destructive effect upon the lower forms of organic animal and vegetable life, in fevers, and particularly the vegetable acids, they but add to the oxidizing processes that are prevailing in the system. To demonstrate our views forcibly to the mind of any practicing physician, let him select from his fever patients a marked case; one in whom the thermometer marks a high figure; one in whom the pulse indicates the most marked features of febrile excitement, and throw aside in his repertory of drugs and diet everything of an acid nature. All sweets and any luscious food, or other kinds of nutriment tending to acidity or fermentation, be laid aside. Let a milk diet be ordered exclusively, and begin with that wonderful antipyretic, bicarbonate of soda, which is harmless for evil, but acts like magic for good. Give it in heroic doses until you have, as it were, saturated your patient. Temperature will then fall, heat abate, the skin will act, the bowels will move, urine will become bland, the bile will flow, absorption will be reinforced, assimilation will slowly but surely begin to take place; the headache and other distressing symptoms will have passed away—and the patient will now speedily recover. As nature abhors a vaccuum, so does disease, or at least fever, abhor neutrality.

In our plan of treatment we accomplish the following indications (and, in fact, what more is to be desired?): Exosmosis throughout the system is re-established, congestions are relieved, germ life or fermentive processes are annihilated, irritation removed and heat dissipated. These are merely the results of acknowledged chemical and physiological action, and they can be nowhere more beautifully demonstrated than in the living organism. Under our system of treatment we have a thousand times witnessed the most wonderful and gratifying results. We may be told, why not use salts of a refrigerative nature, in combination with the vegetable acids, etc.? The acids of these preparations are assimilated, we are told, and the remaining alkali neutralizes the system all the same. This may be true, but we prefer a more positive means of neutralizing the system. We prefer the simpler bicarbonate of soda, a remedy always at hand, and, with anything like moderation, as harmless as wheat flour.

We may be charged with enthusiasm or, perhaps, insanity; these, we admit, are very near relations, but let any physician try my plan for even the short time of forty-eight hours, and he will see things, perhaps, never before dreamt of in his philosophy.

In the treatment, if we find the secretions from the skin reeking with the feverish smell, with the odors, as we may say, of the swill-tub or the vinegar-manufactory, we add to the treatment affusion or sponging with tepid water, containing a large proportion of soda. In fact, the skin, and particularly in the crotches of the arms and thighs, should be well washed, as it were, in the soda solution. This may be made in the proportion of a pound of the bicarbonate to about four or five gallons of water.

In introducing the soda into the system as we propose, we do not, to speak plainly, introduce there a foreign substance, but a something that is natural to it, something that the organism craves, something that is as essential to animal life as the blood itself. Most of the remedies of the fever state are poisons, which, as we believe, in no way accomplish the cure but by checking septic or malarial fermentation, or, in other words, malarial germ growth. Here their efficacy stops; but with the soda it is different. It follows up the retreating disease, and, like a gallant and successful conqueror, presses on the fleeing and demoralized cohorts, and, thus progressing, drives even the scattering guerillas of the fell arm from its own bushes and fastnesses.

Mind, the principle is to saturate, as it were. A half-way plan is like most half-way plans in this life; it is well enough as far as it goes, but it does not go far enough. We use the following formula:

℞ Bicarb. soda.............................℥ ss to ℥ j,

Fld. ext. gelsemium.........................ʒ j,

Spts. nit. ether............................ʒ ss;

Ginger ess.................................ʒ ss,

Peppermint water..........................℥ vijss.

M. Sig. Tablespoonful in half tumbler of water every two or three hours.

In our treatment we avoid all sweets and sours. After convalescence is established, quinia and the mineral acids serve as tonics. But the scrupulous avoidance of every species of vegetable acids and fruits of all kinds are strictly enjoined till health is in every way established. Should the soda operate on the bowels through its combinations with the acids and effete matters in the bowels, this may be stopped by adding to the formula above a drachm of deodorized tincture of opium. In truth, this will be a good addition, as a common thing, to the formula, by which existing irritation is calmed and sleeplessness and delirium avoided.

In fine, we do not flatter ourself we have made a discovery, as many physicians who are successful in the treatment of fever use something of a neutralizing treatment; but they, to a certain extent, are inconsistent by using other things which, more or less, counteract the effect of their neutralizing plan. In spite of all the quinia, etc., you allow your patient the indulgence of his morbid cravings for acids, sweets, etc., and you find him an obstinate case, who will require that you try and try again until failure and on top of failure will ere long disgust both him and you, and you will, perhaps, mutually console yourselves as did Honest Slender in his relations with Mrs. Anne Page: "That there was no great love between you in the beginning, and it pleased heaven to increase it on further acquaintance."

In a word, I have, during my whole course of reading, particularly noticed the effects which have followed neutralizing influences in the treatment of disease. Here we have seen its calming influences in infantile diseases. We have seen the most alarming chronic stages of colliquative diseases of the bowels and stomach gradually mend their way to final recovery. *Per contra*, how often have we seen and fully appreciated the disastrous effects of a treatment with acids, and particularly of the vegetable kind, conjoined with a diet rank with fermentation and *septic* materials.

In conclusion, we would request that physicians will take but a moment's heed of our suggestions and give our plan a trial, and carefully report the comparative results. The medical world is now great on anti-septics, germicides, anti-zymotics, disinfectants, etc., etc. Let them lay down this cumbrous armamentarium, in the treatment of fevers especially, or, better, let them be used as a secondary means in the treatment; then, mark my word for it, there will be inaugurated a new era in medicine more wonderful than dawned upon it after the immortal discovery of Harvey!

By way of addenda, we may say that there is no better remedy in the diarrhœa of typhoid and other fevers than the deodorized tincture opium saturated with tannin. This may be given in the ordinary doses of the tincture of opium.—*Ther. Gazette.*

